# Associations of stunting at 2 years with body composition and blood pressure at 8 years of age: longitudinal cohort analysis from lowland Nepal

**DOI:** 10.1038/s41430-018-0291-y

**Published:** 2018-08-28

**Authors:** Jonathan C. K. Wells, Delan Devakumar, Dharma S. Manandhar, Naomi Saville, S. S. Chaube, A. Costello, David Osrin

**Affiliations:** 1 Childhood Nutrition Research Centre, Population, Policy and Practice Programme, UCL Institute of Child Health, 30 Guilford Street, London, WC1N 1EH UK; 2 UCL Institute for Global Health, London, WC1N 1EH UK; 3 Mother and Infant Research Activities, Kathmandu, Nepal

**Keywords:** Cardiovascular diseases, Nutrition disorders

## Abstract

**Background:**

Stunting remains a very common form of child malnutrition worldwide, particularly in South Asian populations. There is poor understanding of how it develops and how it is associated with subsequent phenotype.

**Subjects**/**methods:**

We used data from a longitudinal cohort of children (*n* = 841) in lowland Nepal to investigate associations of stunting at 2 years with maternal traits and early growth patterns, and with body size and composition, kidney dimensions by ultrasound, lung function by spirometry and blood pressure (BP) at 8 years.

**Results:**

Compared to non-stunted children, children stunted at 2 years came from poorer families and had shorter, lighter mothers. They tended to have higher birth order, were born smaller, and remained shorter, lighter and thinner at 8 years. They had lower leg length, lean and fat masses, smaller kidneys, and reduced lung function (all *p* < 0.0001). These differences persisted with smaller magnitude after adjusting for current height, maternal height and education, family assets and birth order. Stunting was not associated with BP.

**Discussion:**

Stunting developed on an inter-generational timescale in this population and its risk increased with birth order. At 8 years, children stunted at 2 years had deficits in tissue masses and some aspects of physical function that were only partially attributable to their persisting short height and maternal phenotype. This suggests that the early stunting is associated with greater deficits in long-term outcomes than would be expected from the persistent short stature alone.

## Introduction

Until recently, child under-nutrition (low birth weight, reflecting growth restriction in utero; wasting (low weight for height), reflecting acute malnutrition; and stunting (low height for age, reflecting chronic malnutrition)) and overweight/obesity were considered different problems, occurring in different populations and characterised by different risk factors. However, increasing numbers of children in low-income and middle-income countries are reported to be both stunted in early life and overweight or obese at later ages [1–4]. Moreover, some studies suggest that early stunting may contribute to subsequent obesity risk [5, 6].

Initial reports linking stunting and obesity were based on simple anthropometric measures, categorising childhood overweight/obesity by body mass index (BMI). The use of height to assess both stunting and obesity is problematic, with height underestimation increasing the likelihood of being categorised as both stunted and obese [7]. This problem can be circumvented by longitudinal studies, and by obtaining more direct measurements of body composition.

Using such approaches, research provides conflicting evidence regarding the association between stunting and later obesity risk. Studies from South America have linked stunting with elevated body fat levels, mediated by impaired capacity for fat oxidation [5, 6]. However, these findings have not been replicated in other geographical regions [8, 9].

Stunted children are typically already small at birth and have shorter mothers [10–12], indicating an inter-generational contribution. However, it remains unclear that how much this reflects genetic factors rather than chronic exposure to adverse environments [13]. Independent of associations with obesity, stunting may also constrain development of lean tissues and physical function [14, 15].

Both under-nutrition (low birth weight, wasting and stunting) and obesity may contribute independently to non-communicable disease risk, as described in the ‘capacity-load’ model [16]. Child malnutrition may deplete ‘metabolic capacity’, referring to traits that promote cardio-metabolic homoeostasis, while obesity indicates an elevation of ‘metabolic load’, which perturbs homoeostasis [16, 17]. However, as yet, most applications of the capacity-load model have used birth weight as the index of metabolic capacity [18, 19], whereas markers of child malnutrition have received less attention [15, 20].

To date, most of this research has been conducted in African, Caribbean or South American populations. A high proportion of the world’s stunted children live in South Asia [21], and there is particular need to understand the aetiology and long-term consequences of stunting in this geographical region. We investigated longitudinal associations of stunting at 2 years with later body composition and physical function in children from lowland Nepal, who had participated in a randomised trial of micronutrient supplementation during pregnancy. We also explored associations of stunting at 2 years with prior growth patterns, maternal anthropometry, education and family assets, and with birth order.

## Methods

### Original trial

The study was conducted in Dhanusha district, in the lowland Central Terai region of Nepal. The original trial, described in detail elsewhere [22, 23], is registered as an International Standard Randomised Controlled Trial, number ISRCTN88625934.

Briefly, 1200 women attending Janakpur Zonal Hospital for antenatal care were randomly allocated to receive either the UNIMMAP micronutrient supplement (vitamins A, B1, B2, B6, B12, C, D, E and niacin, along with folic acid, iron, zinc, copper, selenium, and iodine; Danish Pharmaceutical Industries Ltd, Denmark) or a control supplement (iron and folic acid). The supplements were taken daily from between 12 and 20 weeks gestation until delivery [22]. Exclusion criteria included multiple pregnancies, foetal abnormalities on obstetric ultrasound, and maternal illness that could compromise pregnancy outcome.

A total of 1069 mothers and infants completed the trial and were assessed at birth. Maternal anthropometry was measured at the time of recruitment. At 2 years, a follow-up was conducted, with weight and height amongst the outcomes assessed [23].

The original trial was approved by the Nepal Health Research Council and by the ethics committee of UCL Institute of Child Health (ICH) and Great Ormond Street Hospital (GOSH), and was undertaken in collaboration with the Nepal Government Ministry of Health. The 8-year follow-up was approved by the same institutions. Verbal and written informed consent was obtained from parents or guardians in local language.

### Eight-year follow-up

Every attempt was made to find children from the original trial using location data from previous follow-up. The follow-up study was powered at 81% to detect a difference of 0.2 standard deviation scores between allocation groups, with a sample size of 400 per group, at 5% significance. Thus, we aimed to re-measure >800 of the original sample.

Anthropometry was measured according to ICH guidelines, adapted from Lohman and colleagues [24] and the WHO Multi-Centre Growth Reference Study [25]. Standing height was measured in duplicate barefoot, with head in the Frankfort plane (Leicester stadiometer, Invicta Plastics, UK), accurate to 0.1 cm. Sitting height was measured in duplicate using a custom-made stool, with base of the spine touching the stadiometer and head in the Frankfort plane. Leg length was calculated as the difference between sitting height and height. Relative leg length was calculated as (leg length/height). Girths of the head, mid-upper arm, chest, waist, hip and mid-upper arm were measured in duplicate using a non-stretchable tape.

Weight and body composition were measured using a Tanita BC-418 scale (Tanita Corp, Japan) accurate to 0.1 kg. Children wore standardised clothing weighing 200 g. Raw impedance was converted to body composition values using a sample-specific isotope-calibration study [26]. Body mass index (BMI) was calculated as weight/height [2], and weight, height and BMI *z*-scores calculated using WHO reference data [25]. Skinfold thicknesses at biceps, triceps, subscapular and suprailiac sites were measured in triplicate using a Harpenden calliper (Assist Creative Resource, Wrexham, UK), accurate to 0.2 mm.

Blood pressure was measured with an Omron M6 electronic monitor (Omron Healthcare Ltd, Japan) with paediatric or adult cuff as required. Measurements followed GOSH guidelines [27]. Blood pressure was recorded after the child had been seated for ≥1 min with legs uncrossed. Two readings were taken one minute apart, with the cuff deflated fully between them. The lowest value was recorded.

Ultrasound measurements of kidney size were taken by a local clinician trained in ultrasonography (Aloka SDD-500 instrumentation, 2–8 MHz convex probe, Aloka Co ltd, Japan), accurate to 1 mm. Maximum renal length and antero-posterior diameter were recorded, ensuring the sinus and parenchyma were visualised using predefined landmarks. Technical error of the mean (TEM) values, calculated from repeat measurements in a 5% subsample, were 0.21 cm (2.6%) and 0.16 cm (1.9%) for right and left kidney lengths, respectively, and 0.14 cm (4.7%) and 0.19 cm (5.7%) for the right and left kidney antero-posterior diameters. There was no systematic bias between first/second measurements.

Lung function was measured using two EasyOne World Spirometers (ndd Medical, Zurich, Switzerland), auto-calibrated before use and alternated fortnightly. American Thoracic Society/European Respiratory Society quality control criteria for spirometry [28], adapted for use in children [29], were used. Parents were requested to bring their children for assessment only if they were well. Three local investigators were trained to conduct spirometry tests. The child performed spirometry wearing a nose clip while seated. All spirographs were interpreted by a clinician (DD) and one in ten over-read by a respiratory physiologist.

Children in whom an illness was suspected were referred to a local paediatrician for more detailed investigation if required, for which the costs were covered. Children whose weight-for-height was <−2 SD or BMI-for-age was <−3 were referred to a local nutrition centre. All children were given a T-shirt, refreshments, and a voucher to be seen by a local paediatrician, external to the research team, with the costs of minor acute treatments covered.

### Conceptual model and statistical analysis

Stunting was defined at 2.5 years of age as height standard deviation score <−2, using WHO child growth reference data [30]. Maternal BMI was used to categorise mothers as underweight (BMI < 18.5 kg/m^2^), overweight (BMI > 23 kg/m^2^), or normal (≤18.5 and ≥ < 23 kg/m^2^). Stunted and non-stunted children were compared for categorical outcomes using chi-square tests, allowing odds ratios and 95% confidence intervals to be calculated, and for continuous outcomes using regression analysis with a dummy variable for stunting, or independent sample *t*-tests. Where Levene’s Test of Homogeneity of Variances was not satisfied, *t*-tests utilised un-pooled variances and a correction to the degrees of freedom.

Relative differences between stunted and non-stunted children were evaluated using ‘sympercents’, calculated as the difference of the natural log-transformed traits multiplied by 100 [31]. This is similar to percentage differences, but avoids the problem that these change in magnitude, depending on which of the groups is used as the reference.

In additional regression analyses, associations of outcomes with early stunting status were adjusted for height at 8 years, in order to assess whether differences between stunted and non-stunted children were entirely explained by persisting differences in height. As stunting might reflect genetic factors, we also adjusted for maternal height. Likewise, long-term associations of stunting with phenotype might reflect continued exposure to poor quality environments, hence we adjusted for maternal education and family asset score. Following preliminary analysis that showed stunting to be more common among those with higher birth order, we adjusted for high birth order status (3rd + born). Finally, since the data derive from a randomised controlled trial, allocation group was also entered in regression models, to test whether it confounded any associations.

To test the capacity-load model, subcutaneous adiposity was categorised as ‘normal’ or ‘high’ using a cut-off for subscapular skinfold of 5.0 mm. This skinfold was selected as it had previously been shown to be the component of adiposity most strongly associated with systolic blood pressure in this population [19]. The association of stunting with systolic and diastolic blood pressure was then assessed in ‘normal’ and ‘high’ subscapular groups, fitting a stunting-subscapular interaction term to test formally for group differences in associations.

## Results

A total of 813 children with data at both 8 years and earlier time points were available for analysis. There was no sex difference in the risk of being stunted, nor was stunting more common in rural than urban environments (Table 1). Stunted children tended to come from families with low levels of economic assets, were less likely to be first-born offspring, and more likely to have high birth order (3+), but were no more likely to be born preterm. Stunted children had reduced likelihood of their mothers being overweight, and increased likelihood of their mothers being thin.

**Table 1:** Differences in early-life traits, maternal traits and early growth trajectory between children stunted or non-stunted at 2 years

	Stunted (*n* = 309)		Non-stunted (*n* = 494)				
	Number	%	Number	%	Odds Ratio^a^	95% CI	*P*-value
Male	157	50.8	258	52.2	0.94	0.71, 1.26	0.6
Rural	171	55.3	255	51.6	1.16	0.87, 1.54	0.3
Low birth weight	106	34.3	64	12.9	3.51	2.47, 4.99	<0.0001
First-born	193	37.7	233	47.2	0.67	0.50, 0.90	0.007
Birth order 3+	108	34.9	113	22.9	1.81	1.32, 2.48	<0.0001
Preterm	24	7.8	28	5.7	1.40	0.80, 2.46	0.2
Maternal BMI < 18.5 kg/m^2^	113	36.7	124	25.1	1.73	1.27, 2.35	<0.0001
Maternal BMI > 23 kg/m^2^	14	4.5	43	8.7	0.50	0.27, 0.93	0.026

	Mean	SD	Mean	SD	Difference^b^	95% CI	*P*-value
Maternal age (y)	21.8	3.8	21.4	3.4	0.4	−0.1, 0.9	0.13
Education (y)	2.7	4.0	5.1	4.7	2.4	−3.1, −1.8	<0.0001
Asset score	1.72	1.20	2.05	1.16	−0.32	−0.49, −0.16	<0.0001
Maternal height (cm)	148.8	5.9	151.9	5.1	3.1	−4.1, −2.6	<0.0001
Maternal BMI (kg/m^2^)	19.3	2.3	20.0	2.3	0.7	−1.0, −0.3	<0.0001
Gestational age (weeks)	39.2	1.6	39.6	1.6	−0.4	−0.6, −0.1	0.002
Birth weight (kg)	2.61	0.39	2.88	0.41	−0.27	−0.33, −0.21	<0.0001
Birth length (cm)	48.2	2.5	49.3	3.1	−1.1	−1.5, −0.7	<0.0001
Birth head girth (cm)	33.3	2.3	33.9	2.2	−0.6	−0.9, −0.2	<0.0001
Age at follow up at 2.5 years	2.6	0.4	2.5	0.3	0.0	−0.0, 0.1	0.3
Weight (kg)	9.5	0.8	11.5	1.2	−2.0	−2.1, −1.8	<0.0001
Length (cm)	81.2	3.8	85.3	4.5	−4.2	−4.7, −3.6	<0.0001
BMI (kg/m^2^)	14.5	1.1	15.8	1.4	−1.3	−1.5, −1.2	<0.0001
Head girth (cm)	45.9	1.4	46.8	1.4	−0.9	−1.1, −0.7	<0.0001

^a^Odds ratio computed from Chi-square test

^b^Difference computed by independent samples *t*-test

Table 1 also presents maternal data. Maternal age at enrolment did not differ between stunted and non-stunted children. Stunted children had mothers with lower educational attainment, lower height and lower BMI. Stunted children had slightly shorter average gestations than non-stunted children, and had smaller size (weight, length, head circumference) at birth. At 2.5 years of age, they remained smaller for all anthropometric outcomes. There was no difference in age at follow-up by stunting status.

First-borns were 151 (95% CI 95, 207) g lighter than later-borns at birth, though they showed no difference in birth length. By 2 years, they had 0.27 (0.12, 0.42) greater weight *z*-score, and 0.20 (0.06, 0.34) greater length *z*-score. At 8 years, they remained 0.8 (0.3, 1.3) kg and 1.6 (0.8, 2.4) cm taller than later-borns. Birth order showed no continuous association with birth length, maternal BMI or maternal height, whereas birth weight showed an n-shaped association with parity (Supplementary online Fig. 1).

**Fig. 1:**
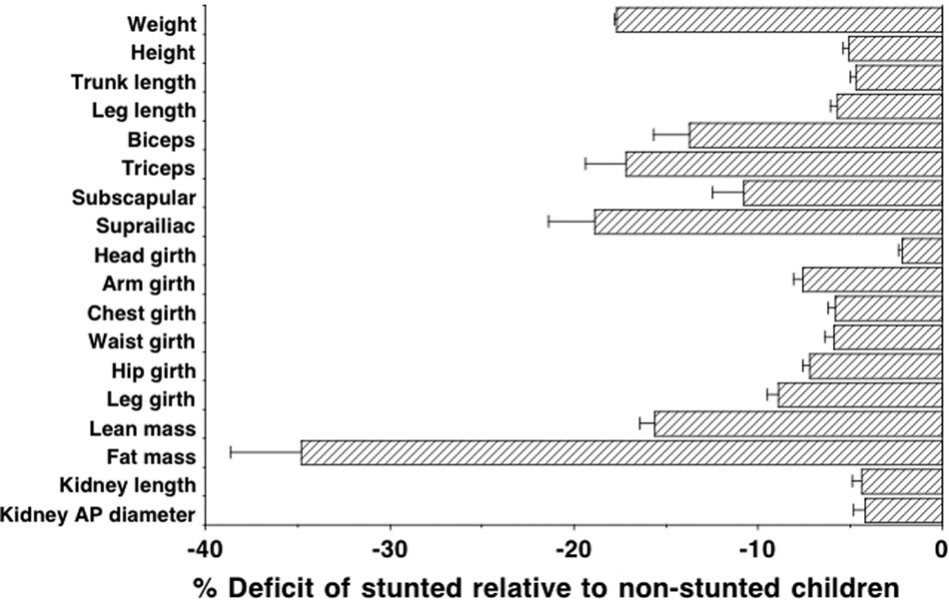
Deficits in individual body components of stunted children, relative to non-stunted children, expressed in sympercents. Error bars are standard error of the group difference

Those stunted at 2 years had an odds ratio of 8.2 (95%CI 5.8, 11.6) for remaining stunted at 8 years, and remained smaller for every anthropometric outcome, having shorter leg and trunk lengths, smaller skinfold thicknesses, and smaller body girths (Table 2), whilst also having lower variance for some outcomes. Their lower relative leg length indicates altered body proportions, independent of their short stature. Stunted children were on average 0.1 year younger than non-stunted children at follow-up, but this had negligible effect on differences in anthropometry, body composition or physical function. Figure 1 illustrates the deficits and standard error by somatic outcome, illustrating that they were smallest for head girth and kidney dimensions, intermediate for body girths, and largest for lean mass, fat mass and peripheral skinfolds.

**Table 2:** Differences in age and anthropometry at 8 years between children stunted or non-stunted at 2 years

	Stunted (*n* = 309)		Non-stunted (*n* = 494)				
	Mean	SD	Mean	SD	Difference^a^	95%CI	*P*-value
Age (years)	8.4	0.3	8.5	0.4	−0.1	−0.1, −0.0	0.004
Weight (kg)	18.1	1.9	21.6	3.3	−3.5	−3.9, −3.2	<0.0001
Height (cm)	116.8	4.9	123.0	5.3	−6.2	−6.9, −5.4	<0.0001
BMI (kg/m^2^)	13.2	0.9	14.2	1.4	−1.0	−1.2, −0.8	<0.0001
Trunk length (cm)	62.3	2.3	65.3	2.7	−3.0	−3.3, −2.6	<0.0001
Leg length (cm)	54.6	3.1	57.8	3.1	−3.2	−3.6, −2.8	<0.0001
Relative leg length (%)	46.7	1.3	46.9	1.0	−0.3	−0.4, −0.1	0.001
Biceps (mm)	3.4	0.9	3.9	1.5	−0.5	−0.7, −0.3	<0.0001
Triceps (mm)	6.4	1.7	7.6	2.8	−1.2	−1.5, −0.8	<0.0001
Subscapular skinfold (mm)	4.4	1.0	4.9	1.6	−0.6	−0.8, −0.4	<0.0001
Supra-iliac skinfold (mm)	4.8	1.6	5.8	2.8	−1.0	−0.3, −0.7	<0.0001
Head girth (cm)	48.6	1.4	49.7	1.4	−1.0	−1.2, −0.8	<0.0001
Mid-upper arm girth (cm)	15.2	0.9	16.4	1.4	−1.2	−1.4, −1.0	<0.0001
Chest girth (cm)	53.7	2.5	56.9	3.5	−3.1	−3.6, −2.7	<0.0001
Waist girth (cm)	47.3	2.8	50.2	4.0	−2.9	−3.4, −2.5	<0.0001
Hip girth (cm)	54.8	2.7	58.9	3.9	−4.0	−4.5, −3.6	<0.0001

^a^Difference computed by independent samples *t*-test

Table 3 describes differences in body composition and physiological outcomes. In unadjusted analyses, stunted children had lower values for lean mass, fat mass and kidney dimensions, as well as lean and fat mass indices. These differences broadly remained in multiple regression analyses, after correction for height at 8 years, maternal height and education, high parity and trial allocation group, though the difference in kidney length was no longer significant (*p* = 0.061). There was no difference in blood pressure between the groups in unadjusted or adjusted models.

**Table 3:** Differences in body proportions, body composition, blood pressure and lung function at 8 years between children stunted or non-stunted at 2 years

	Stunted (*n* = 309)		Non-stunted (*n* = 494)				
Unadjusted outcomes	Mean	SD	Mean	SD	Difference^a^	95% CI	*P*-value
Lean mass (kg)	15.4	1.7	18.0	2.2	−2.6	−2.9, −2.3	<0.0001
Fat mass (kg)	2.4	0.9	3.4	1.7	−0.9	−1.1, −0.7	<0.0001
Lean mass index (kg/m^2^)	11.3	0.9	11.9	0.9	−0.6	−0.7, −0.5	<0.0001
Fat mass index (kg/m^2^)	1.8	0.7	2.2	1.0	−0.4	−0.5, −0.3	<0.0001
Kidney length (cm)	7.91	0.60	8.27	0.51	−0.36	−0.44, −0.28	<0.0001
Kidney AP diameter (cm)	3.06	0.24	3.19	0.24	−0.12	−0.16, −0.90	<0.0001
Systolic BP (mmHg)	97.6	7.6	98.5	7.7	−0.8	−1.9, 0.2	0.12
Diastolic BP (mmHg)	61.1	7.8	61.5	7.9	−0.3	−1.5, 0.8	0.5
FEV1 ¥	1.09	0.17	1.27	0.20	−0.17	−0.20, −0.15	<0.0001
FVC ¥	1.23	0.18	1.45	0.23	−0.23	−0.26, −0.20	<0.0001

Adjusted outcomes					Difference^b^	95% CI	*P*-value
Lean mass (kg)					−0.8	−1.0, −0.5	<0.0001
Fat mass (kg)					−0.2	−0.5, −0.0	0.046
Kidney length (cm)					−0.07	−0.15, 0.00	0.061
Kidney AP diameter (cm)					−0.06	−0.10, −0.02	0.001
Systolic BP (mmHg)					−0.9	−2.2, 0.4	0.18
Diastolic BP (mmHg)					−0.5	−1.8, 0.8	0.4
FEV1 ¥					−0.03	−0.06, −0.01	0.005
FVC ¥					−0.06	−0.09, −0.03	<0.0001

^a^Difference by independent samples *t*-test in unadjusted analyses

^b^Difference from multiple regression analysis, adjusting for height at 8 years, high birth order (3rd+), maternal height, maternal education, family assets and trial group

¥ *n* = 305 and 492

Stunted children had lower markers of lung function. In crude analyses, stunted children had −15.0 (95% CI −12.8, −17.3) % lower FEV1, and −16.8 (−14.6, −19.1) % lower FVC. These differences were largely reduced in magnitude if adjusted for current height and other confounders, but remained significant (FEV1: ∆ = −3.4 (−5.4, −1.3) %; FVC: ∆ = −4.9 (−6.8, −2.9) %).

**Fig. 2:**
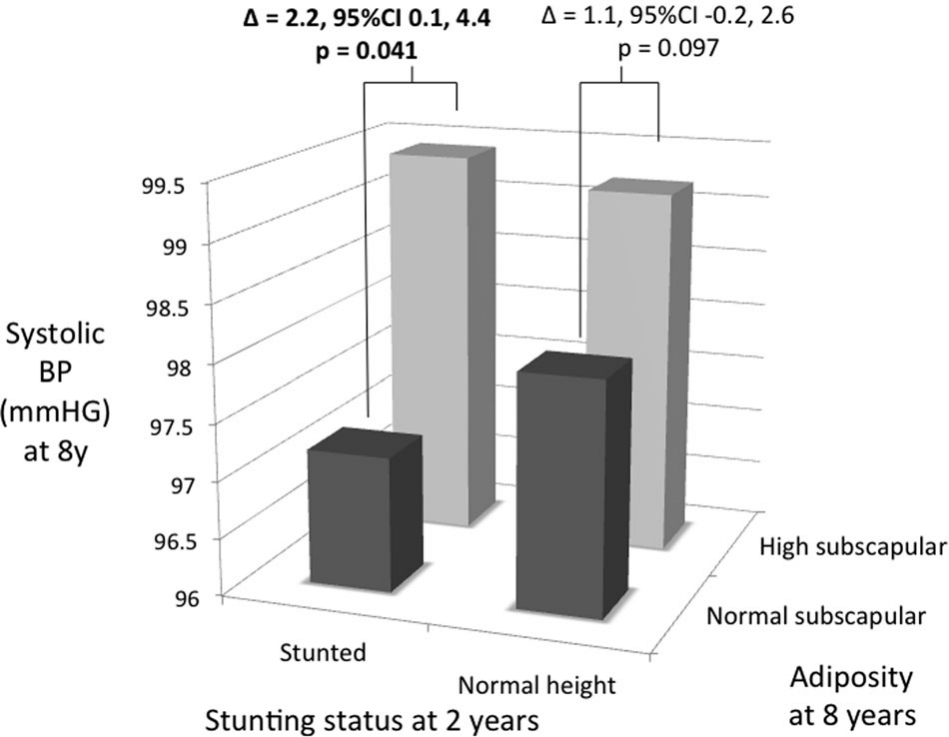
Association of high adiposity with systolic blood pressure at 8 years, stratified by presence/absence of stunting at 2 years. Children were categorised as having ‘normal’ or ‘high’ subscapular skinfold at 8 years, using a cut-off of 5.0 mm. Relative to normal adiposity, high adiposity was associated with higher BP in stunted children, but not in non-stunted children, however this group difference did not achieve statistical significance

When stratified by ‘high’ versus ‘normal’ levels of subscapular skinfold, stunted children showed a larger difference than non-stunted children in systolic BP (Fig. 2). Among both stunted and non-stunted children, systolic BP increased in association with elevated adiposity. Whereas this increase was 2.2 (0.1, 4.4) mmHG in the stunted children, it was only 1.1 (−0.2, 2.6) mmHG in the non-stunted children, however this group contrast was not itself significant. Equivalent differences for diastolic blood pressure did not reach significance.

Trial allocation group was significant in models of birth weight and size at 2 years, but not at 8 years, and its inclusion in regression models for outcomes at 8 years had negligible effect on the coefficient of stunting (data not shown).

## Discussion

Our analysis of longitudinal data from lowland Nepal indicate that stunting represents an inter-generational process. Children stunted at 2 years came not only from poorer families, but also from mothers who were shorter and thinner than those of non-stunted children, and were less likely to be first-borns and more likely to have high birth order (3+). The process of poor linear growth began in utero and was predicted by both social and biological maternal factors.

By 8 years, stunted children had not recovered in their growth, and showed absolute deficits in all parameters of body size and composition, whilst also having relatively shorter legs. The differences in body composition and physical function remained significant, though of smaller magnitude, after adjusting for current stature and maternal or family traits associated with stunting. These analyses indicate that, beyond linear growth, stunting is associated with additional deficits in somatic development and physical function.

This is the first study to investigate the association between stunting and the size of specific organs in childhood. Differences between groups in kidney dimensions were substantially reduced after adjusting for current height and its correlates, consistent with previous research demonstrating a close association between organ growth and linear growth during childhood. Nevertheless, small differences in kidney dimensions remained even after this size adjustment, suggesting that stunting may constrain organ growth over and above its constraint on linear growth. Likewise, stunting was associated with poorer lung function even after taking body size into account, though the magnitude of the association was small. In a recent study from Malawi, severe-acute malnutrition in early postnatal life showed little long-term association with lung function [32].

While stunting was not associated with overt differences in BP, stunted children showed a greater increase in systolic BP than non-stunted children among those with elevated subscapular skinfold. However, this group difference was not itself statistically significant. Higher BP in stunted than non-stunted overweight women has previously been reported in Brazil [33], suggesting that obesity might be more ‘toxic’ in those previously stunted [16, 17, 19]. Further follow-up of our cohort is needed to ascertain whether the trend identified here amplifies with age.

Our findings differ from some studies in South American populations, which reported higher levels of body fat and elevated obesity risk among stunted children [6], mediated by impaired fat oxidation [5]. However, these findings were not replicated in several other studies from other geographical regions. In a study from Peru, height and adiposity were positively correlated in lowland children, but inversely correlated in highland children [34]. In our analysis, all measures of adiposity were reduced in stunted children and many of these differences remained apparent even after correcting for their shorter height. In this population, stunting thus manifests as a marker of broader and persistent malnutrition.

An important finding was the association of stunting with birth order. Although first-borns typically have smaller size at birth [35–38], this is often followed by catch-up growth that rapidly ‘overshoots’ the growth deficit, resulting in taller height than later-borns from childhood [37, 39, 40]. In Brazil, this catch-up was mostly achieved by 8 months and fully achieved by 12 months [35], suggesting it is mediated by internal growth acceleration rather than family size. We found a similar pattern, with first-borns significantly heavier and taller than later-borns at 2 and 8 years of age. Higher birth order therefore emerges as a risk factor for stunting, but we could not attribute this to shorter length at birth. However, stunted children were more likely to have underweight mothers, and maternal overweight was associated with a lower risk of stunting.

Exactly why stunting is associated with poorer physiological function is unclear. Stunting may signal continued exposure to poverty, food insecurity and other stresses, and may also incorporate genetic effects [41]. Nevertheless, we found adverse associations of stunting that were independent of both maternal height and markers of poverty. Recent studies suggest that stunting is associated with prior wasting (unpublished data). The notion that short stature conceals more overt nutritional constraints at earlier time points may therefore explain why adverse long-term associations are still apparent after controlling for differences in height per se. However it is also possible that our measures of family circumstances, collected at baseline, do not adequately index variability in childhood exposure to deprivation.

Our data derived from a randomised controlled trial, in which the intervention exerted modest effects on size at birth and 2 years, though not at 8 years. The adverse associations of stunting remained evident after adjustment for trial allocation, and indeed at 2 years the coefficient for stunting was substantially greater than that for trial group.

In summary, we found that early stunting is associated with reduced height, leg length, lean mass and kidney size, and that these associations are stronger than expected from the constraint of linear growth per se. Stunting thus has important long-term health implications in the geographical region where it is most prevalent.

## Electronic supplementary material

Supplementary Figure 1
