# Supplementary figures and images for: Associations of stunting at 2 years with body composition and blood pressure at 8 years of age: longitudinal cohort analysis from lowland Nepal
Source: Eur J Clin Nutr. 2018 Aug 28;73(2):302–10. doi: 10.1038/s41430-018-0291-y (PMC6368558; doi:10.1038/s41430-018-0291-y)

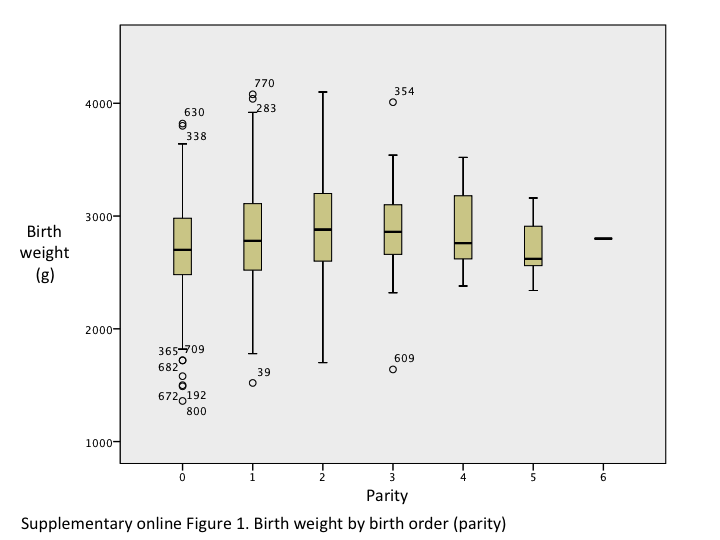

Supplement: Supplementary file 1 — Supplementary Figure 1 [file 41430_2018_291_MOESM1_ESM.tif]
